# Ischemic postconditioning prevents surge of presynaptic glutamate release by activating mitochondrial ATP-dependent potassium channels in the mouse hippocampus

**DOI:** 10.1371/journal.pone.0215104

**Published:** 2019-04-12

**Authors:** Shohei Yokoyama, Ichiro Nakagawa, Yoichi Ogawa, Yudai Morisaki, Yasushi Motoyama, Young Su Park, Yasuhiko Saito, Hiroyuki Nakase

**Affiliations:** 1 Department of Neurosurgery, Nara Medical University, Kashihara, Japan; 2 Department of Neurophysiology, Nara Medical University, Kashihara, Japan; Albany Medical College, UNITED STATES

## Abstract

A mild ischemic load applied after a lethal ischemic insult reduces the subsequent ischemia–reperfusion injury, and is called ischemic postconditioning (PostC). We studied the effect of ischemic PostC on synaptic glutamate release using a whole-cell patch-clamp technique. We recorded spontaneous excitatory post-synaptic currents (sEPSCs) from CA1 pyramidal cells in mouse hippocampal slices. The ischemic load was perfusion of artificial cerebrospinal fluid (ACSF) equilibrated with mixed gas (95% N_2_ and 5% CO_2_). The ischemic load was applied for 7.5 min, followed by ischemic PostC 30 s later, consisting of three cycles of 15 s of reperfusion and 15 s of re-ischemia. We found that a surging increase in sEPSCs frequency occurred during the immediate-early reperfusion period after the ischemic insult. We found a significant positive correlation between cumulative sEPSCs and the number of dead CA1 neurons (r = 0.70; p = 0.02). Ischemic PostC significantly suppressed this surge of sEPSCs. The mitochondrial K_ATP_ (mito-K_ATP_) channel opener, diazoxide, also suppressed the surge of sEPSCs when applied for 15 min immediately after the ischemic load. The mito-K_ATP_ channel blocker, 5-hydroxydecanoate (5-HD), significantly attenuated the suppressive effect of both ischemic PostC and diazoxide application on the surge of sEPSCs. These results suggest that the opening of mito-K_ATP_ channels is involved in the suppressive effect of ischemic PostC on synaptic glutamate release and protection against neuronal death. We hypothesize that activation of mito-K_ATP_ channels prevents mitochondrial malfunction and breaks mutual facilitatory coupling between glutamate release and Ca^2+^ entry at presynaptic sites.

## Introduction

In the heart and brain, a brief sublethal ischemic load prior to lethal ischemia induces tolerance to the subsequent ischemic insult, a phenomenon known as ischemic preconditioning [1]. Ischemic preconditioning provides powerful protection against ischemia-reperfusion injury in both the heart and nervous system [2–6]. A previous study demonstrated that ATP-dependent potassium (K_ATP_) channel opener-induced chemical preconditioning is involved in neuroprotection [7]. Although preconditioning is an effective treatment for protection against cerebral and myocardial damage, its clinical use is limited, because the onset of an ischemic stroke or cardiac infarction is extremely difficult to predict. In contrast, the onset of reperfusion following symptomatic ischemia is relatively easy to predict. Therefore, the concept of postconditioning (PostC) in the brain and heart has been proposed [8–10]. PostC consists of several repeated cycles of brief re-occlusion and reperfusion, which is started early in the reperfusion period after a lethal ischemic load [8]. Zhao and colleagues first reported that PostC reduces cerebral ischemia–reperfusion injury [10].

Excessive glutamate release is an important event that mediates ischemia-reperfusion injury in the brain [11, 12]. Elevation of extracellular glutamate levels causes depolarization of the neuronal membrane, and the intracellular Ca^2+^ concentration is then increased due to activation of voltage-dependent Ca^2+^ channels and NMDA receptor channels [13]. Glutamate release and cytosolic Ca^2+^ increase via a positive feedback loop, because cytosolic Ca^2+^ facilitates glutamate release. Therefore, suppressing glutamate release is a critical factor in protection of the brain from ischemia–reperfusion injury. Nevertheless, no reports have described the electrophysiological mechanisms of PostC that mediate glutamate release.

K_ATP_ channels are present on the cell membrane and the inner membrane of mitochondria in various organs including brain. K_ATP_ channels play an important role in hypoxia in in vivo experiments [14, 15]. A previous whole-cell recording study in gerbil hippocampus revealed that inhibition of the marked increase in glutamate release during ischemia by preconditioning is mediated by K_ATP_ channels [7, 16]. A recent report suggests that K_ATP_ channels are also involved in the effect of PostC on tolerance to ischemic damage [17]. Among the many mechanisms for both preconditioning and PostC, opening of the mitochondrial ATP-dependent potassium (mito-K_ATP_) channels has received much attention [18–21]. In addition, involvement of the mito-K_ATP_ channel pathway in cerebral anesthetic-induced PostC has recently been highlighted [20, 21]. Therefore, chemical PostC with a mito-K_ATP_ opener may be an effective therapeutic strategy for treating acute cerebral ischemic stroke [9, 10, 22]. However, the relationship between mito-K_ATP_ channels and neuroprotection induced by PostC remains to be elucidated [23].

In this study, we investigated excitatory post-synaptic currents (EPSCs) from CA1 pyramidal cells using whole-cell patch-clamp recordings, and found that ischemic PostC suppressed synaptic glutamate release during the reperfusion period. We further examined the involvement of mito-K_ATP_ channels in ischemic PostC using 5-hydroxydecanoate (5-HD) (a blocker of mito-K_ATP_ channels) and diazoxide (DZX) (an opener of mito-K_ATP_ channels).

## Materials and methods

### Slice preparation

Wild-type C57BL/6JJcl mice weighing 18–22 g and aged 28–35 days were used. All experimental procedures were performed in accordance with the Guidelines for Proper Conduct of Animal Experiments (Science Council of Japan, 2006) and approved by the animal care and use committee of the Nara Medical University. Animals were anesthetized with isoflurane and decapitated. The brain was rapidly removed and submerged in an ice-cold solution composed of (mM): 230 sucrose, 2.5 KCl, 1.25 NaH_2_PO_4_, 10 MgSO_4_, 0.5 CaCl_2_, 25 NaHCO_3_, and 10 glucose, equilibrated with mixed gas (95% O_2_ and 5% CO_2_). The hippocampal formation was cut into horizontal slices 350 μm thick in this solution using a vibratome (Vibratome 1000 Plus 102, Pelco International). The slices were then incubated in artificial cerebrospinal fluid (ACSF) composed of (mM): 125 NaCl, 2.5 KCl, 1.25 NaH_2_PO_4_, 1 MgCl_2_, 2 CaCl_2_, 25 NaHCO_3_, and 10 glucose, equilibrated with the same mixed gas at 32 °C for at least 1 h, and then maintained in ACSF at 27 °C.

### Whole-cell recordings

CA1 pyramidal neurons were identified visually by shape with a water immersion objective (LUMPlanFL40XW/IR2, Olympus) on an upright microscope (BX50WI, Olympus) equipped with an infrared TV camera (C2741, Hamamatsu Photonics). Conventional whole-cell patch–clamp recordings from CA1 pyramidal neurons were obtained using an EPC-9 amplifier (HEKA). Patch pipettes were prepared from borosilicate glass micropipettes that were filled with an internal solution of (mM): 141 K gluconate, 4 KCl, 2 MgCl_2_, 2 Mg-ATP, 0.3 Na-GTP, 10 HEPES, and 0.2 EGTA. The pH was adjusted to 7.3 with KOH. A liquid junction potential (13 mV) was corrected. After an on-cell patch configuration was obtained under visual control, the whole-cell configuration was established by applying negative pressure until the cell membrane within the tip of the electrode ruptured. The neuron was voltage-clamped at a holding potential of −70 mV. All neurons studied had a seal resistance above 1.5 GΩ. Series resistance (i.e., electrode resistance plus access resistance) was monitored every 30 s during the whole-cell recording. When the series resistance exceeded 25 MΩ, the recording was discarded. Spontaneous EPSCs (sEPSCs) were continuously recorded from one pyramidal neuron in each slice before, during, and after the ischemic insult. Current data were low-pass filtered at 2 kHz (3 dB, four-pole Bessel filter) and sampled at a 5-kHz digitization frequency. In all experiments, picrotoxin (50 μM, Wako Pure Chemical) was added to the external solution to suppress GABA_A_ receptor-mediated postsynaptic currents.

### Ischemic insult and PostC

The slice was placed in a recording chamber and immobilized with a U-shaped platinum wire and nylon fibers. The recording chamber was continuously perfused with gas-saturated ACSF at a flow rate of 2.0 ml/min at 32 ± 2 °C. The volume of the chamber was about 800 μL in the recording condition, and an objective lens was immersed in the chamber. Ischemic insult was induced to the slice by perfusion with glucose-free ACSF in which glucose was replaced with isosmotic sucrose; the ACSF was equilibrated with 95% N_2_ and 5% CO_2_. We first determined the optimal ischemic loading time for whole-cell recordings of EPSCs from hippocampal pyramidal cells in this experimental system. Ischemic loadings of 7, 7.5, and 8 min were examined, and 7.5 min was optimal because we observed an irreversible insult after 8 min and an insufficient insult after 7 min. Ischemic loading started after baseline recording for more than 10 min. The PostC procedure included three cycles of 15 s of ischemic perfusion with an intermittent 15 s of reperfusion. The ischemic PostC started 30 s after the onset of the reperfusion period.

### Cell staining

To investigate the relationship between sEPSC accumulation and neuronal cell death, we visualized dead cells in hippocampal slices following the ischemic insult using two membrane-impermeable fluorescent dyes for nuclear staining, propidium iodide and SYTOX-blue. The slice was incubated in ACSF containing 3 μM propidium iodide for 15 min less than 45 min before electrophysiological recording. The slice was loaded 7.5 min ischemic insult, re-perfused with ACSF for 20 min, and then transferred into an incubation chamber. After incubation in ACSF for 3 h at 32°C, the slice were stained with 6 μM SYTOX-blue. Dead cells were observed using a confocal microscope (C2plus, Nikon). To detect cell that have died before ischemic insult, propidium iodide was excited at 561 nm, and the red fluorescent emission was band-pass filtered between 552 and 617 nm. SYTOX-blue was excited at 408 nm, the blue fluorescent emission was band-pass filtered between 417 and 477 nm. The number of dead cells showing only blue fluorescent in the CA1 region was counted.

### Experimental design

**Fig 1:**
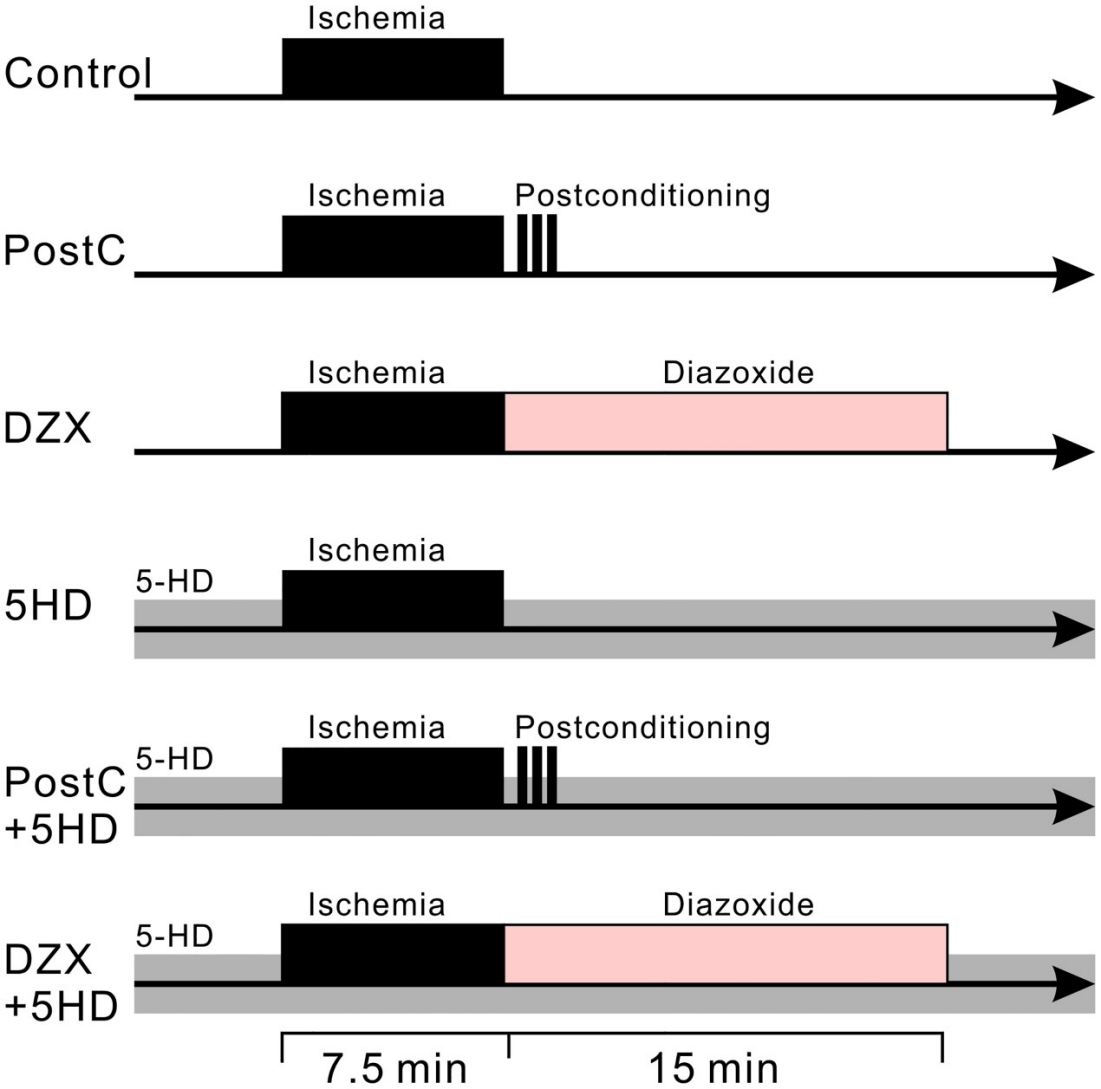
Diagrams representing the perfusion protocol for experimental groups. Ischemic perfusion is shown as a black band. Gray and faded-pink bands indicate administration of 5-HD and diazoxide to the bath solution, respectively. Electrophysiological recordings started 5 min before the ischemic perfusion and lasted up to 20 min after the reperfusion.

We also examined the effects of chemical PostC with DZX (500 μM) and treatment with 5-HD (200 μM) on ischemia-induced increases in synaptic glutamate release. Hippocampal slices were randomly assigned to one of six groups (Fig 1): (1) control group: the slices were subjected to 7.5 min of ischemic perfusion and then reperfusion with ACSF for 20 min; (2) PostC group: after 7.5 min of ischemic perfusion and 30 s of reperfusion, the slices were exposed to the PostC procedure and then reperfused with ACSF; (3) DZX group: after 7.5 min of ischemic perfusion, the slices were perfused with ACSF containing DZX for 15 min, and then with normal ACSF; (4) DZX and 5-HD group: both normal and glucose-free ACSF contained 5-HD (200 μM) throughout the recording period. The perfusion schedule was the same as for the DZX group; (5) 5-HD group: both normal and glucose-free ACSF contained 5-HD. The perfusion schedule was the same as for the control group; (6) PostC and 5-HD group: both normal and glucose-free ACSF contained 5-HD throughout the recording period. The perfusion schedule was the same as for the PostC group.

### Data analysis

sEPSCs were recorded and quantified digitally with in-house software (Mscan) that automatically selects events and allows rejection or acceptance of individual events. Current events with a rapid negative (inward) deflection of more than 6 pA followed by a gradual decay were detected as EPSCs. Spurious events were manually rejected before further analysis. All quantitative results were expressed as means ± standard error of the mean (n = the number of cells recorded). Analysis of variance was used to compare the data. Statistical analyses were performed using Sigma Stat software (Jandel Scientific, Erkrath, Germany). Statistical significance was determined at the level of p < 0.05. Significant effects were further tested with a post-hoc multiple comparison test (Tukey-Kramer method).

## Results

### Excessive accumulation of sEPSCs tended to increase the number of death neurons

**Fig 2:**
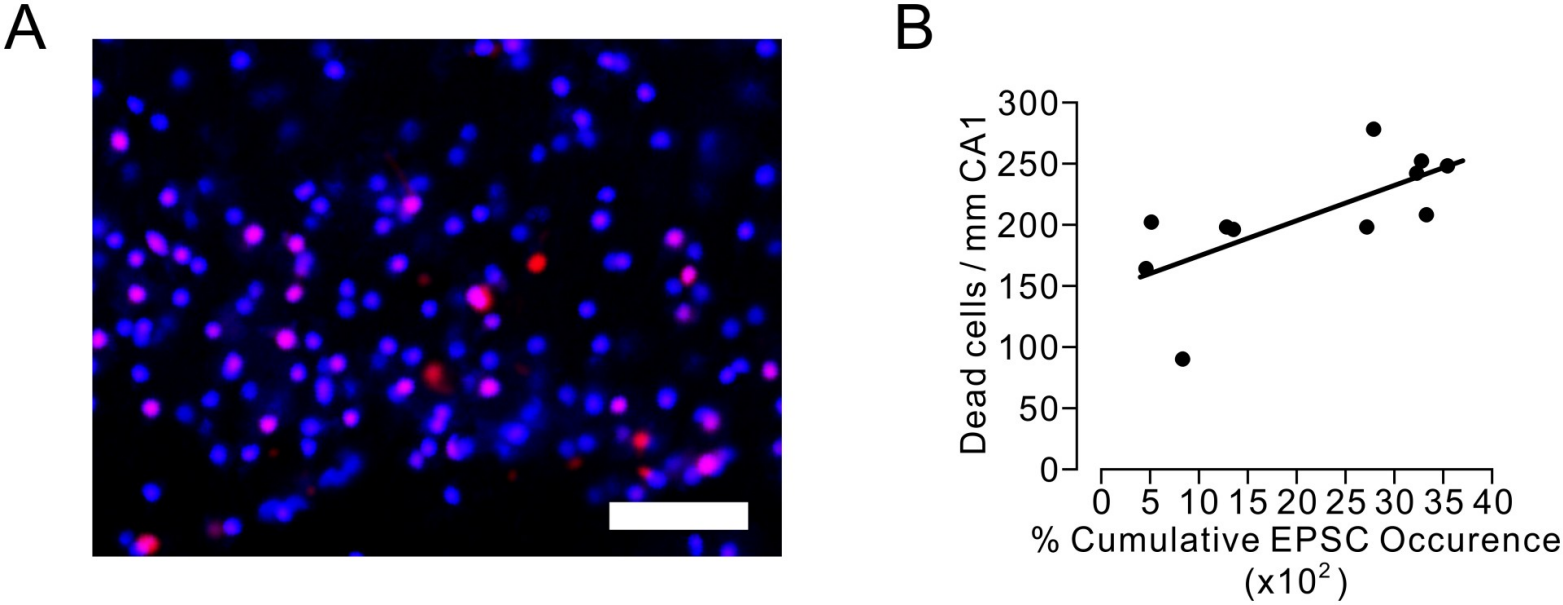
Correlation between cumulative EPSC occurrence and the number of cells died due to ischemic insult in hippocampal CA1 area. A. Microscopic images of CA1 area showing nuclei of dead cells. Magenta-colored cells stained with both propidium iodide and SYTOX-blue were considered to have died before electrophysiological recording. Blue-colored cells stained with only SYTOX-blue were assumed to have died due to ischemia-reperfusion injury. Scale bars = 50 μm. B. Two-dimensional scatter plot of cumulative EPSC occurrence and dead cell number per 1 mm of CA1 area. A positive correlation between cumulative EPSC occurrence and surviving cell number was statistically significant (Pearson’s correlation coefficient r = 0.70, n = 11, Student’s t = 2.96, p = 0.016). The line represents an estimated linear regression.

We examined whether the accumulation of synaptic glutamate release would cause neuronal death at the immediate-early stage of the post-ischemic reperfusion period. Because neuronal death is caused not only by ischemia-reperfusion injury but also by the slice preparation procedure in this experiment, we used two membrane-impermeable dyes for nuclear stain with different fluorescent wave lengths to detect exclusively the cells that have died during the period from the electrophysiological recording to 3 h after ischemic insult of 7.5 min (Fig 2A). We found a significant positive correlation between the percent cumulative EPSCs and the number of dead cells (Pearson’s correlation coefficient r = 0.70, n = 11, Student’s t = 2.95, p = 0.016) (Fig 2B). This positive correlation indicates that excessive accumulation of sEPSCs tended to cause neuronal death, even during the immediate-early stage of the post-ischemic reperfusion period.

### Ischemic PostC suppressed the surge of sEPSCs

**Fig 3:**
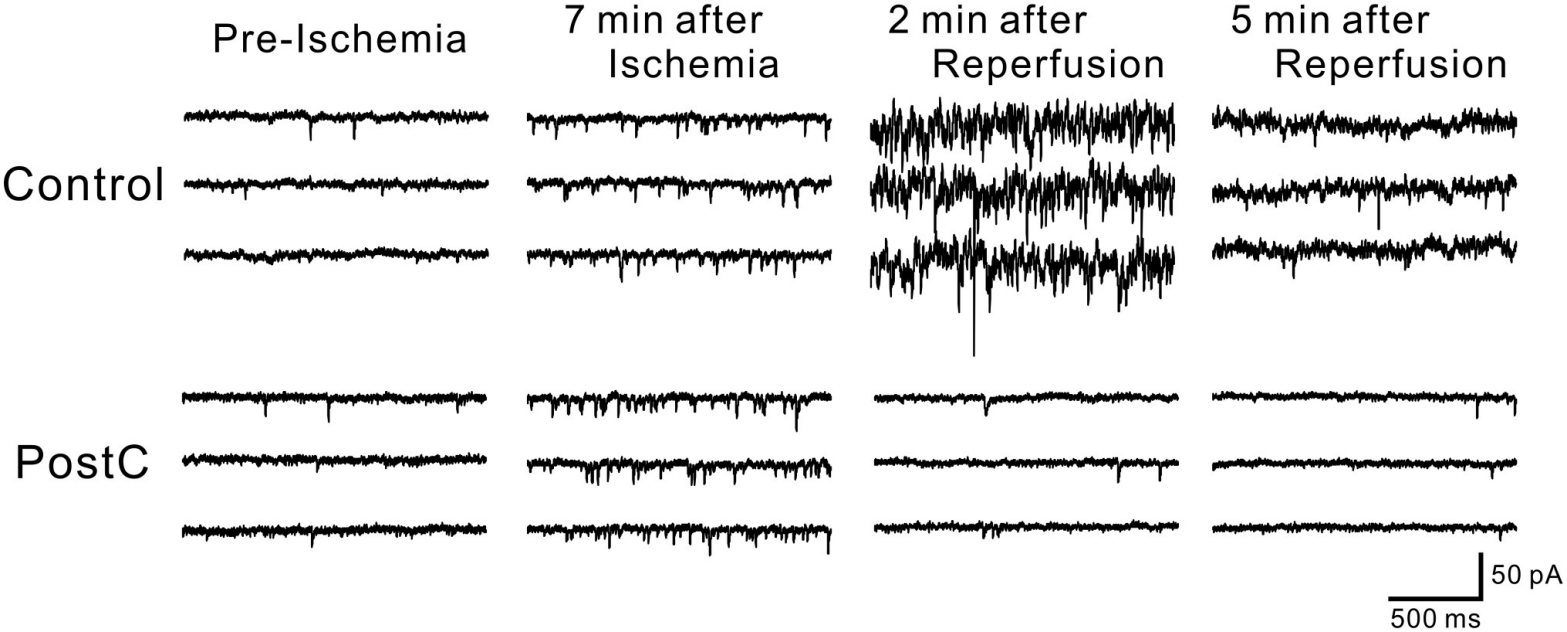
Representative traces of EPSCs for Control (upper) and PostC (lower) groups during pre-ischemic, ischemic and reperfusion periods. In each trace, EPSCs caused by synaptic glutamate releases are observed as transient downward deflections (inward currents). For both Control and PostC groups, the occurrence of EPSCs began to increase apparently 7 min after ischemic perfusion. In the traces of Control group, an explosive increase in EPSC frequency was seen 2 min after reperfusion. On the other hand for PostC group, increased occurrence of EPSCs was receding to pre-ischemic level immediately after reperfusion.

**Fig 4:**
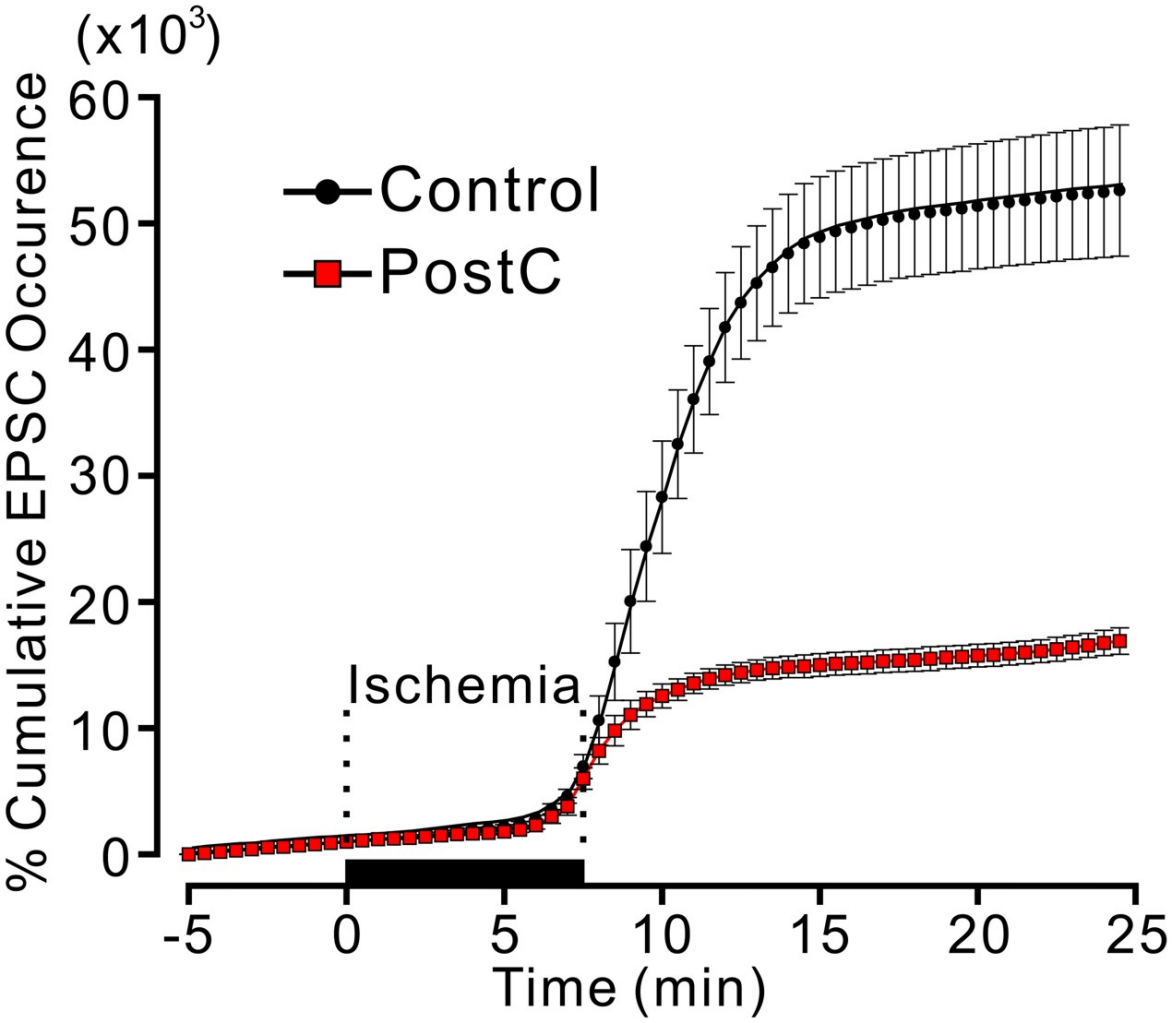
Time course of cumulative EPSC occurrence for Control and PostC groups. In each line plot, the marker and error bar show mean and standard error of mean (SEM), respectively. The cumulative value of EPSC occurrence is expressed in percent of the total number of EPSC occurrence during pre-ischemic 5 min. The large majority of EPSCs occurred during 5 min after the reperfusion for the both groups.

With an ischemic load for 7.5 min, the sEPSC frequency gradually increased in the latter half of the ischemic perfusion period, then increased drastically after reperfusion, and then gradually decreased. Ischemic PostC suppressed this drastic increase in the sEPSC frequency (Fig 3). We statistically analyzed the cumulative number of EPSCs, which were expressed as a percent of EPSCs during the 5 min before ischemia. The percent cumulative EPSCs 20 min after the onset of ischemic perfusion in the ischemic PostC group was significantly lower than that in the control group (PostC group: 15.7 ± 0.91 × 10^3^%; control group 51.3 ± 5.0 × 10^3^%, p < 0.01) (Fig 4).

### Chemical PostC in the presence of the mito-K_ATP_ channel opener, DZX, also suppressed the surge in sEPSCs

**Fig 5:**
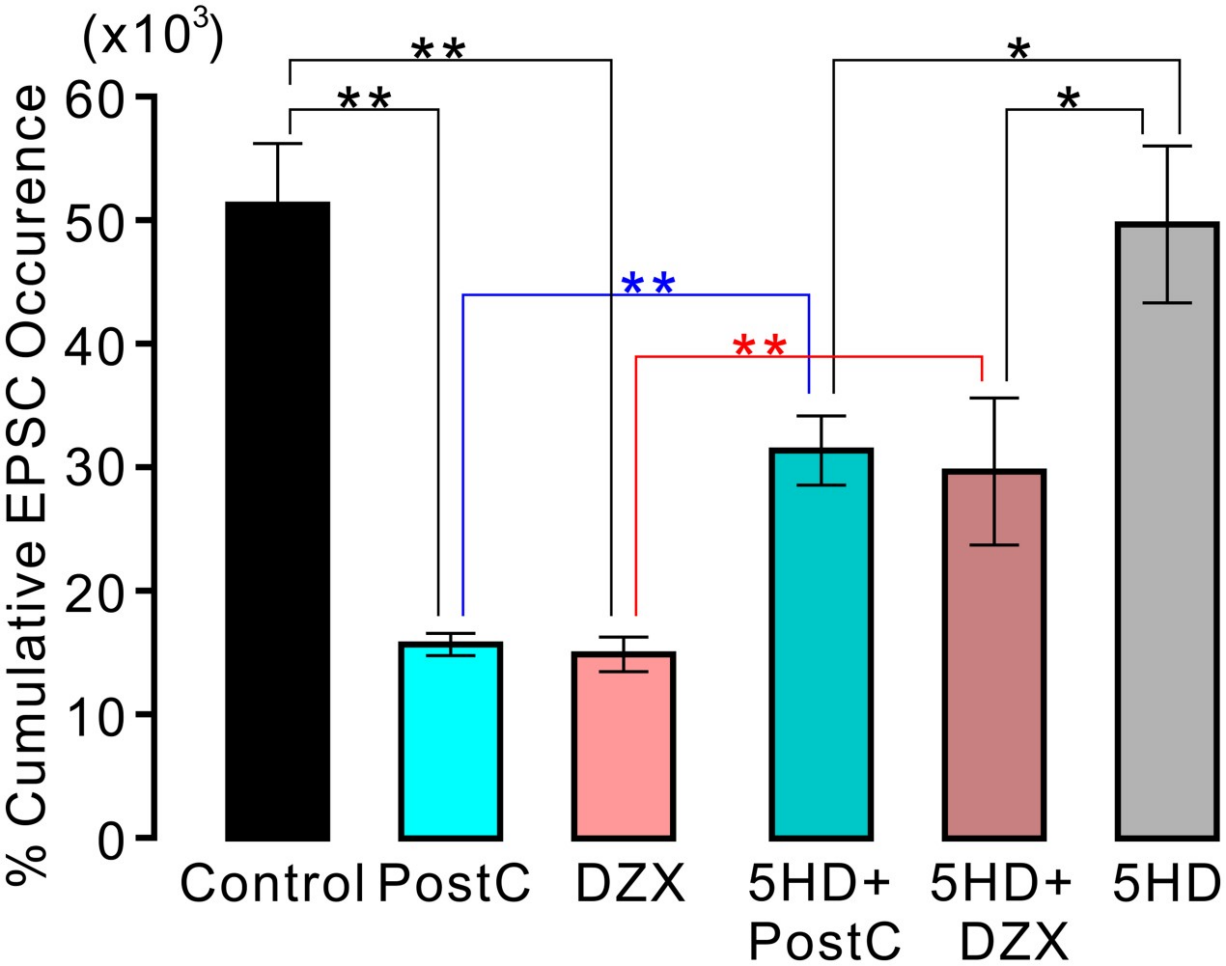
Effects of postconditioning and diazoxide application on cumulative EPSC occurrence. Each vertical rectangle and error bar indicate percent cumulative EPSC occurrence at 20 min after onset of ischemic perfusion (12.5 min after reperfusion) and SEM, respectively. For all the groups, the number of data is ten. Asterisks indicate significant difference in post-hoc pairwise comparisons (**: p < 0.01, *: p < 0.05).

The increase in sEPSCs during the reperfusion period was also suppressed by the application of 500 μM DZX (Fig 4). The percent cumulative EPSCs 20 min after the onset of ischemic perfusion in the DZX group was significantly lower than that in the control group (DZX group 14.9 ± 1.4 × 10^3^%; control group 51.3 ± 5.0 × 10^3^%, p < 0.01). The suppressive effect of DXZ application on sEPSC frequency was comparable to that of ischemic PostC. We found no significant difference in the percent cumulative sEPSCs between the PostC and DZX groups (Fig 5).

### The mito-K_ATP_ channel blocker, 5-HD, attenuated the suppressive actions of ischemic and chemical PostC on the surge of sEPSCs

The presence of a mito-K_ATP_ channel blocker (5-HD) in ACSF did not affect the drastic increase in sEPSC frequency during the reperfusion period. The percent cumulative EPSCs 20 min after the onset of ischemic perfusion in the 5-HD group did not differ significantly from that in the control group (5-HD group: 49.7 ± 6.3 × 10^3^%, p < 0.05). However, the presence of 5-HD in the ACSF weakened the suppressive effects of ischemic PostC and chemical PostC with DZX (Fig 5). The percent cumulative EPSCs in the PostC + 5-HD group was significantly higher than that in the PostC group (PostC + 5-HD group: 29.7 ± 5.9 × 10^3^%; PostC group: 15.7 ± 0.91 × 10^3^%, p < 0.01). Similarly, the percent cumulative EPSCs in the DZX + 5-HD group was significantly higher than that in the DZX group (DZX + 5-HD group 31.4 ± 2.8 × 10^3^%; DZX group 14.9 ± 1.4 × 10^3^%, p < 0.01). Nevertheless, the percent cumulative EPSCs in the PostC + 5-HD and DZX + 5-HD groups was significantly lower than that in the 5-HD group (p < 0.05, Fig 5).

## Discussion

In the present electrophysiological study using mouse hippocampal slices, we found that reperfusion after an ischemic insult caused a surge of synaptic glutamate release. Ischemic PostC suppressed this surge. Excessive glutamate release during the post-ischemia-reperfusion period tended to promote neuronal death, even early during the post-ischemic period, demonstrating that PostC in this study may have had a neuroprotective effect. Chemical PostC with the mito-K_ATP_ channel opener, DZX, also suppressed the surge of synaptic glutamate release. In addition, the mito-K_ATP_ channel blocker, 5-HD, attenuated the suppressive actions of ischemic and chemical PostC on the surge of synaptic glutamate release. These results suggest that the opening of mito-K_ATP_ channels is involved in suppression of the surge of glutamate release and in protection against neuronal death.

### The surge of glutamate release may be a critical event that triggers processes leading to brain damage

The protective effect of ischemic PostC depends on the number of ischemia-reperfusion cycles, the durations of the ischemic insult and reperfusion, and the time of onset of PostC. Gao et al. examined these parameters using a bilateral common carotid artery occlusion model in rats, and demonstrated a significantly reduced infarct volume by PostC that consisted of three cycles of 10 s of reperfusion and 10 s of occlusion that started immediately after the ischemic insult [24]. Several researchers have used a unilateral medial carotid artery occlusion model and reported that PostC that consisted of three cycles of 30 s of reperfusion and 30 s of occlusion effectively reduces the infarct volume [25–27]. Although six cycles of 30 s of reperfusion and 30 s of occlusion reduce inflammation and infarct [28], most ischemic PostC procedures were completed within 200 s after the end of the ischemic insult. Furthermore, delayed or long-lasting PostC cycles do not reduce the infarct size [24]. These findings indicate that a certain critical event that triggers the subsequent processes responsible for neuronal death occurs within 200 s after the ischemic insult, and that PostC procedures suppress this critical event. Demonstrating such an event through in vivo experiments may be difficult. In the present in vitro mouse hippocampal slice study, the slices received a 7.5-min ischemic insult with deoxygenated glucose-free ACSF and were then reperfused with oxygenated ACSF. We found that a drastic increase in synaptic glutamate release occurred immediately after the ischemic insult and lasted about 5 min. In addition, PostC that consisted of three cycles of 15 s of ischemia and 15 s of oxygenated perfusion applied 30 s after the ischemic insult suppressed the drastic increase in synaptic glutamate release, and showed a neuroprotective effect. We thus speculate that this surge of glutamate release represents the critical event that triggers subsequent catastrophic processes.

### Ischemic PostC reduces the excessive accumulation of extracellular glutamate

Glutamate is the main excitatory transmitter in the brain, and glutamate release from presynaptic terminals during and after an ischemic insult causes depolarization of neurons, which enhances the influx of extracellular Ca^2+^ through voltage-dependent Ca^2+^ channels and NMDA receptors [29]. Excessive accumulation of cytosolic Ca^2+^ modifies the activity of a number of enzymes (proteinases, phospholipases, nitric oxide synthases, and others), which eventually results in neuronal cell death due to damage to membranes, nuclei, and other cellular organelles [30, 31]. Therefore, lowering the extracellular glutamate level is an essential treatment for protection against ischemia-reperfusion injury in the brain. Delayed ischemic PostC applied 2 days after ischemia reduces the glutamate concentration in the cortex and hippocampal regions [32]. Glial cell, in particular astrocyte, has been known to release glutamate via exocytosis or through tranporters and ion channels in response to ischemia [32–35]. Since astrocyte serves for the clearance of glutamate released from neurons in normal condition, glutamate release by astrocytes may result in extremely high glutamate level at synaptic site, which might be involved in the surge of glutamate release observed in this study. Histochemical studies indicate that ischemic PostC accelerates extracellular glutamate clearance by upregulating glutamate transporter 1 and glutamine synthetase expression in glial cells [23, 36]. These mechanisms possibly contribute to the neuroprotective effects of ischemic PostC.

In the present study, we found another mechanism of ischemic PostC for reducing extracellular glutamate. The extracellular glutamate concentration was previously thought to be elevated rapidly after the onset of ischemia and to decline ordinarily following reperfusion. Our results demonstrated that rapid recovery of pO_2_ and the glucose concentration after reperfusion did not simply reduce the extracellular glutamate level but reversed the surge of synaptic glutamate release. Ischemic PostC applied immediately after the ischemic perfusion suppressed the surge of synaptic glutamate release, which should reduce the extracellular glutamate accumulation. Because the ischemic PostC procedure in this study was completed 105 s after the ischemic perfusion, the action of PostC cannot be continuous. Rather, ischemic PostC likely acts as a switch to terminate the surging glutamate release. Excessive glutamate can depolarize cell membrane to activate voltage dependent Ca^2+^ channels. Therefore, glutamate release from presynaptic terminals may be facilitated by Ca^2+^ entry. We suggest that ischemic PostC may interrupt mutual facilitatory coupling between the Ca^2+^ entry to presynaptic terminals by depolarization and the glutamate releasing mechanism.

Since synaptic glutamate release cannot occur in cardiac tissue, one could wonder whether the surging glutamate release that we observed is a critical cause for the ischemia-reperfusion injury process or not. The surge of glutamate release may reflect some abnormal intracellular event triggering cell-injuring sequence. This abnormal event could be an increase in cytosolic Ca^2+^, although Ca^2+^ accumulated during ischemic period should be rapidly cleared by ATP-dependent pumps after the recovery of pO_2_ and glucose concentration. This paradoxical cytosolic Ca^2+^ elevation could also occur in cardiac myocytes.

### Role of mito-K_ATP_ channels in ischemic PostC

The mito-K_ATP_ channel opener, DZX, has neuroprotective actions [37, 38]. DZX can mimic the neuroprotective effects of ischemic preconditioning [39] and PostC [17], and pretreatment with the mito-K_ATP_ channel blocker, 5-HD, nullifies these effects. In addition, halogenated inhalational anesthetics such as isoflurane or sevoflurane exert neuroprotective effects by working as mito-K_ATP_ channel openers, and their action is inhibited by 5-HD application [20, 21]. These previous studies have proven that the opening of mito-K_ATP_ channels is an essential process that is part of the mechanism of ischemic PostC; this was confirmed in this study. The present study furthermore suggests a concrete role for opening of mito-K_ATP_ channels in suppression of the surge of glutamate release.

It has been shown that DZX dose-dependently inhibit succinate dehydrogenase (complex II) activity to reduce succinate oxidation in cardiac myocyte [40–42]. In this study, we used a high dose of DZX (500 μM) to apply DZX at sufficient concentration to the cytosol of neurons located even in deep portion of the slice. Since it is probable that DZX can inhibit sufficiently inhibit succinate dehydrogenase activity in this study, the inhibited succinate dehydrogenase activity can bring about suppressive effect of DZX on the surge. In addition, it has been reported that DZX inhibits succinate dehydrogenase activity without changing the electrical potential of mitochondrial inner membrane, and that metabolized 5-HD provides a substrate for β oxidation [43, 44]. Whether the proposition that mito-K_ATP_ channels are not involved in the actions of diazoxide and 5-HD is applicable to brain neuronal cells remains to be determined. Nevertheless, it seems solid that diazoxide suppresses rapid recovery of ATP synthesis during reperfuion period immediately after ischemia. It is, therefore, possible that the injury of mitochondria and/or rapid elevation of ATP concentration immediately after ischemia might trigger cell membrane depolarization, Ca^2+^ entry and the surge of glutamate release. The time course of cytosolic Ca^2+^ and mitochondrial electrical potential during the ischemia-perfusion period should be investigated in future studies.

## Conclusions

The present electrophysiological study demonstrated that synaptic glutamate release was drastically increased during the immediate-early reperfusion period after an ischemic insult, and that ischemic PostC suppressed this surge of glutamate release. Chemical PostC with DZX equivalently reduced the increase in glutamate release. These actions were weakened by pre-application of 5-HD, suggesting that the opening of mito-K_ATP_ channels is essential for suppression of the surge in glutamate release.

## Supporting information

S1 Dataset (XLSX)
